# Cortico-Cortical Interactions Influence Binocularity of the Primary Visual Cortex of Adult Mice

**DOI:** 10.1371/journal.pone.0105745

**Published:** 2014-08-26

**Authors:** Susanne Dehmel, Siegrid Löwel

**Affiliations:** 1 Department of Systems Neuroscience, Bernstein Fokus Neurotechnologie, Johann-Friedrich-Blumbach-Institut für Zoologie und Anthropologie, Georg-August-Universität Göttingen, Göttingen, Germany; 2 Sensory Collaborative Research Center 889, Georg-August-Universität Göttingen, Göttingen, Germany; University of Oxford, United Kingdom

## Abstract

Electrophysiological studies have revealed that a large proportion of the mouse primary visual cortex (V1) receives input also from the ipsilateral eye. This is surprising as most optic nerve fibers cross at the optic chiasm in mice. Inactivating V1 of one hemisphere has recently demonstrated a strong contribution of one hemisphere's activity on binocularity of single units and visually evoked potentials of V1 in the other hemisphere of young rats and of single units in young adult mice. Here we used intrinsic signal optical imaging to quantitatively study the influence of cortico-cortical connections on the magnitude of neuronal activation in the entire binocular zone of adult mouse V1. We simultaneously measured V1-activity of both hemispheres in adult C57BL/6J mice before and after blocking sensory-driven activity in one hemisphere with muscimol. In V1 contralateral to the inactivation, ipsilateral eye evoked activity was reduced by on average 18% while contralateral eye evoked activity did not change. Our results clearly show that cortico-cortical interactions exert a global amplification of ipsilateral eye evoked activity in adult mouse V1.

## Introduction

**Figure 1:**
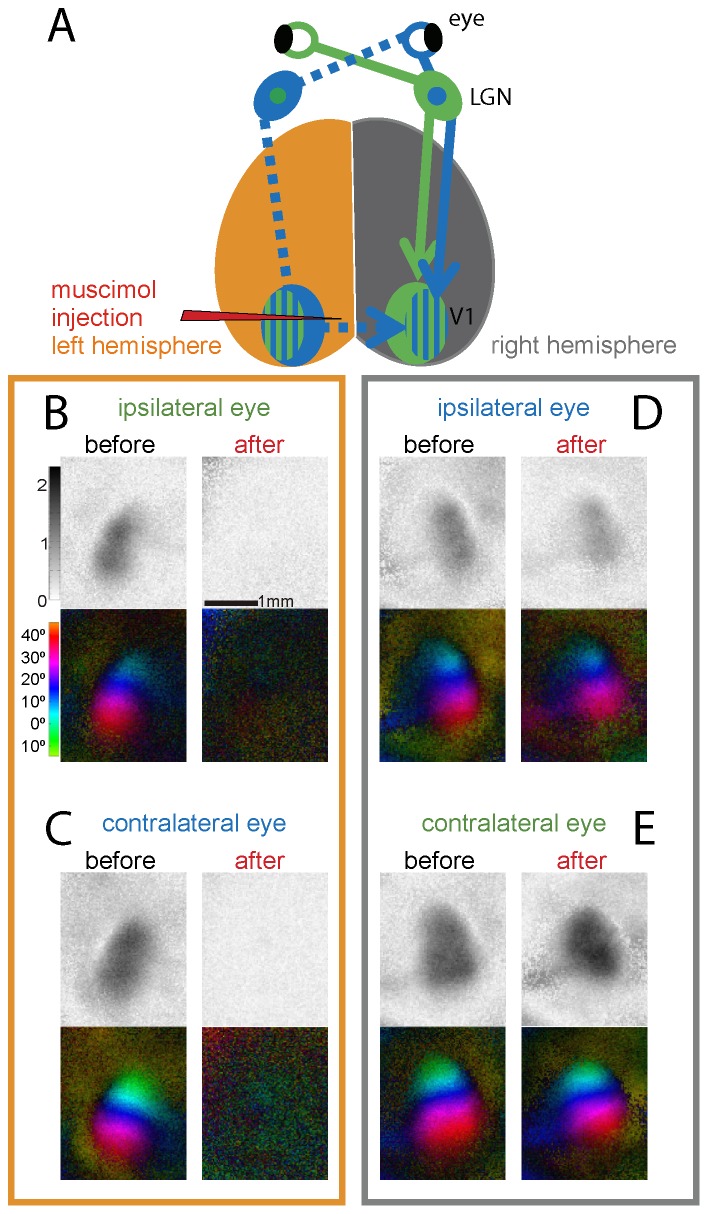
Muscimol-inactivation of the left hemisphere reduced ipsilateral eye-evoked activity in the right V1 of adult mice. (A) Schematic diagram of ipsilateral and contralateral input pathways to the right non-injected V1, and the location of the muscimol-injection. For clarity, inputs to the left V1 are not shown. (B–E) V1-activation after ipsilateral and contralateral eye stimulation in the left (B, C) and right V1 (D, E), visualized by *in vivo* optical imaging of intrinsic signals before (black, left columns) and after injection of muscimol into the left hemisphere (red, right columns). Response magnitude maps (top rows) and polar maps of retinotopy (bottom rows) of a representative example illustrating both the blockade of activity in the muscimol-injected cortex (B,C) and weakening of ipsilateral, but not contralateral eye evoked activity in right V1 (D,E).

One important characteristic of neurons in the mammalian primary visual cortex (V1) is their binocularity, i.e. they get activated by both eyes. In rodents, contralateral eye input dominates V1 and ipsilateral eye input is restricted to the binocular part of V1 [1], [2], [3]. While the majority of optic nerve fibers crosses at the optic chiasm in mice [4], [5] electrophysiological studies revealed a preponderance of binocular cells in V1 [1], [2], [6], [7]. Therefore additional pathways besides the classical retinothalamocortical projections must play a role in determining binocularity and especially in transmitting ipsilateral eye input into V1. One possible pathway is the corpus callosum connecting the two hemispheres (Fig. 1A). In fact, cortico-cortical interactions have recently been shown to play an important role in ocular dominance and its plasticity in juvenile rats (P16-31): Inactivating one hemisphere decreased ipsilateral eye evoked responses in V1 of the opposite hemisphere and thus had a strong influence on ocular dominance [8], [9]. Furthermore, in juvenile rats with monocular deprivation, the cortico-cortical influence suppressed the deprived eye responses [8]. In addition, in vivo extracellular recordings in PD35-90 mice have recently shown that the ipsilateral eye's response was reduced by removing the callosal input in a total of nine cells [10]. It is – however – not yet known whether the callosal input has a global and homogeneous effect on ipsilateral eye activity throughout mouse V1 or not. To this end, we used optical imaging of intrinsic signals as a minimally invasive method to visualize neuronal activity simultaneously in both V1 to allow a quantitative estimate of cortico-cortical influence on the population response of V1 neurons. We could show that cortico-cortical interactions exert a global amplification of ipsilateral eye evoked activity in the primary visual cortex of adult mice.

## Methods

### Animals

Female C57BL/6J mice were obtained from the mouse colony of the Georg-August-Universität Göttingen, Germany. The mice were between 84 and 101 d of age at the day of optical imaging and were raised in standard cages on a 12-h light/dark cycle, with food and water available ad libitum. All experimental procedures were approved by the local government (Niedersächsisches Landesamt für Lebensmittelsicherheit und Verbraucherschutz, registration number 339-42502-04012/0868).

### Surgical preparations

Surgical preparations for optical imaging were described previously [11], [12], [13]. Briefly, after initial box-anesthesia with 2% isofluorane in a mixture of O_2_/N_2_O (50/50), the animals received atropine (0.3 mg) and dexamethasone (0.2 mg) subcutaneously and chlorprothixene (0.2 mg) intramuscularly. Lidocaine (2% xylocain jelly) was applied to all incisions. The animals were placed in a stereotaxic frame. Silicon oil was applied to protect the cornea from drying. The animals' body temperature was maintained at 37°C and subcutaneous electrocardiograph leads were attached to monitor the heart rate throughout the experiment. Anesthesia was maintained with 0.6%–0.8% isoflurane in a mixture of O_2_/N_2_O (50/50). The skin above the skull was incised above V1 of both hemispheres; imaging was performed through the intact skull. Low-melting point agarose (2.5% in NaCl) and a glass cover-slip were placed over the exposed area.

### Optical imaging of intrinsic signals and visual stimuli

Mouse V1-responses were recorded using the imaging method developed by Kalatsky and Stryker [11], [12]. A temporally periodic stimulus was continuously presented to the animal and the cortical response at the stimulus frequency was extracted by Fourier analysis. Optical images of intrinsic signals were obtained using a 1M30 CCD camera (Dalsa) controlled by custom software. Using a 50 mm×50 mm tandem lens configuration (Nikon, Inc.), we imaged a cortical area of 12.3×12.3 mm^2^, thus recording signals from both hemispheres simultaneously. The surface vascular pattern and intrinsic signal images were visualized with illumination wavelengths set by a green (550±10 nm) or red (610±10 nm) interference filter, respectively. After acquisition of a surface image, the camera was focused 600 µm below the cortical surface. An additional red filter was interposed between the brain and the CCD camera. Frames were acquired at a rate of 30 Hz temporally binned to 7.5 Hz and stored as 512×512-pixel images after spatial binning of the camera image. The stimulation monitor was centered in front of the mouse at a distance of 25 cm (Asus, 60 Hz refresh rate, 1680×1050, 51×29 cm, 92×60° visual angle). Drifting horizontal bars were generated by a G450 board (Matrox Graphics, Inc.), controlled by custom software. A full field horizontal bar (92° width, 2° height) was presented at a temporal frequency of 0.125 Hz to obtain activity and retinotopic maps of V1 of both hemispheres simultaneously.

### Data analysis

**Figure 2:**
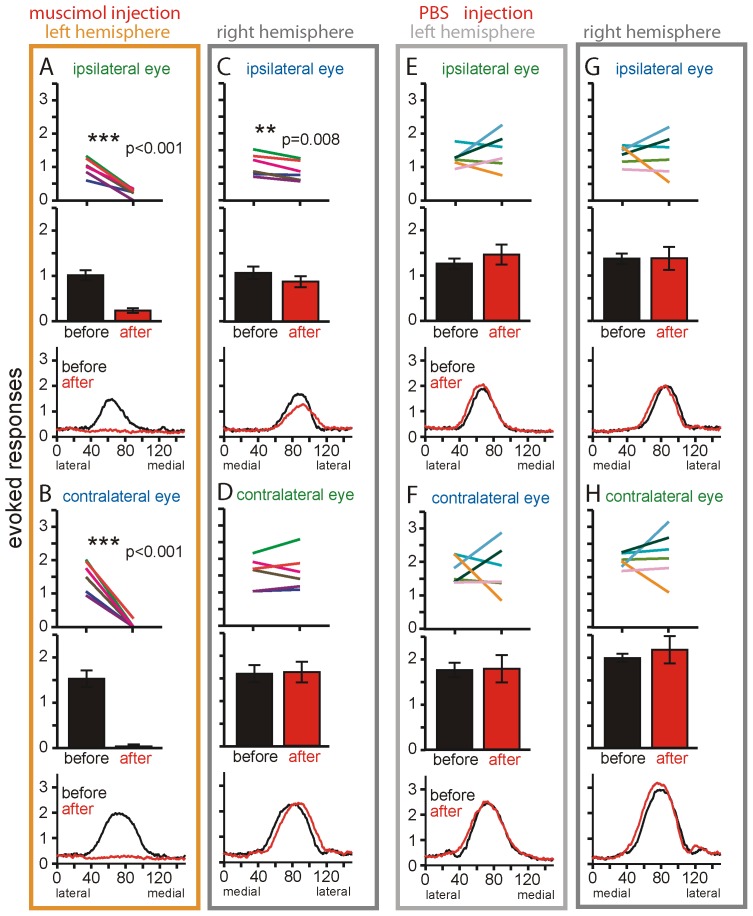
Quantitative analyses of the effect of muscimol- and control PBS-injection on V1-activation. Data of the the injected (left) and non-injected (right) V1 are shown. (A, B) Muscimol reliably blocked both ipsilateral (A) and contralateral eye evoked activity in the left V1 (B), reduced ipsilateral but not contralateral eye evoked V1-activity in the right V1 (C, D), while PBS-injection in a control group of animals had no significant effect (E–H). Evoked responses shown as line plots from single animals (top; each line represents data from one animal before/after the muscimol- or PBS-injection, p of paired t-tests are given), bar plots with group averages (middle plots) and mediolateral profiles (bottom plots, X-axis in 150 pixels equaling 3600 µm) before (black) and after injection (red).

Visual cortical maps were recorded with binocular stimulation followed by monocular stimulation of the left eye and right eye. Each map was derived from responses to 5 min. stimulation with an upwards moving bar and 5 min. stimulation with a downwards moving bar. Two maps per stimulus condition (binocular, left eye monocular, right eye monocular) were averaged. Visual cortical maps were calculated from the acquired frames by Fourier analysis to extract the signal at the stimulation frequency using custom software [12]. While the phase component of the signal is used for the calculation of retinotopy (color coded), the amplitude component represents the intensity of neuronal activation (expressed as fractional change in reflectance ×10^4^, for details, see [11]). In polar maps, hue encodes visual field position (retinotopy) and lightness encodes the magnitude of the visual responses. Recorded maps were thresholded at 30% of the maximum pixel intensity. All pixels with intensities above threshold were averaged and displayed as bar plots with mean±SEM of evoked responses. Activity profiles (Fig. 2) correspond to medio-lateral cross-sections at a middle position through the maps and plot fractional change in reflectance ×10^4^ of the pixels along the cross-section.

### Muscimol injections

After localizing V1 by intrinsic signal optical imaging with binocular stimulation, a small hole (diameter 0.5 mm) was drilled through the skull without injuring the dura and optical imaging was continued to record baseline activity maps evoked by ipsi- and contralateral eye stimulation. Then muscimol (0.25 µl, 10 mM in phosphate buffered saline, PBS) or PBS (control group of animals) was injected through the hole at a depth of 500 µm below the cortical surface. The injection was performed with a glass pipette (10 µm tip diameter) attached to a Nanoliter 2000 Injector (WPI Inc., USA). Injection sites were located medially to V1 (on average 550 µm medial to the medial border of the recorded binocular activity map; mediolateral extent of the contralateral eye map: 1400 µm) and caudally from the rostral border of the recorded map (on average 800 µm; rostrocaudal length of the contralateral eye map: 1800 µm). Injections of radiographic muscimol (0.05–0.4 µl of 8.7 mM) into the basal forebrain and thalamus revealed a diffusion path length of up to 3 mm depending on the diffusion time, volume and concentration of the injected solution [14]. Muscimol penetrates the meninges when applied to the dura, but it does not diffuse through the white matter and into the thalamus [15]. In our experiments, the visual responses of the injected hemisphere were blocked compared to the responses of the non-injected hemisphere. We therefore assume that the diffusion of muscimol followed gravity, spreading in lateral-ventral direction into large parts of the injected hemisphere, most likely inactivating visual, auditory and somatosensory areas. Optical imaging was continued approximately 20 min. after the muscimol/PBS-injection.

### Statistical analyses

All before muscimol/PBS versus after muscimol/PBS comparisons of response amplitudes were done with paired t-tests. Levels of significance were set as *p<0.05; **p<0.01; ***p<0.001. Data are represented as mean±SEM.

## Results

### Muscimol injection in the left V1 reduced ipsilateral eye evoked activity in the right V1

Injection of muscimol significantly reduced ipsilateral as well as contralateral eye evoked V1-activity in the injected hemisphere (ipsilateral eye (i): 1.02±0.11 (mean±SEM) before and 0.24±0.05 after muscimol-injection, paired t-test, p<0.001; contralateral eye (c): 1.54±0.19 before and 0.04±0.04 after muscimol-injection, paired t-test, p<0.001; Figs. 1B, C). This decrease was observed in all animals (n = 6, line plots in Figs. 2A, B). In contrast, injections of the carrier (PBS) in a second group of animals did not significantly change either ipsilateral or contralateral eye evoked activity (i: 1.27±0.11 before and 1.47±0.22 after PBS-injection, paired t-test, p = 0.39; c: 1.75±0.16 before and 1.78±0.30 after PBS-injection, paired t-test, p = 0.94; Figs. 2E,F). Monitoring sensory-driven activity in the left V1 therefore proved its successful inactivation enabling us to study the contribution of cortico-cortical interactions to sensory-driven activity of the non-injected right hemisphere.

Muscimol-inactivation of the left hemisphere significantly reduced ipsilateral eye evoked activity in the right V1 of all animals (n = 6) (Fig. 1D): Average V1-activity induced by visual stimulation of the ipsilateral (right) eye was reduced from 1.07±0.14 to 0.88±0.12 after muscimol-inactivation, corresponding to an average reduction of 18% (paired t-test, p = 0.008, Figs. 2C). In contrast, V1-activity evoked by stimulation of the contralateral eye was not significantly influenced (Fig. 1E) (before 1.59±0.19 and 1.62±0.23 after muscimol-injection, paired t-test, p = 0.76, Fig.2D). In a control group, PBS-injections did not cause significant changes of either ipsi- or contralateral eye evoked activities in the non-injected right hemisphere (i: before 1.37±0.11 and 1.38±0.25 after PBS-injection, paired t-test, p = 0.98; c: 1.99±0.09 and 2.17±0.30 after PBS-injection, paired t-test, p = 0.56; Fig. 2G,H).

In order to compare our data to previous single unit measurements at different mediolateral positions [8] we additionally plotted mediolateral profiles of visually evoked activities before and after muscimol/PBS-injections (Fig. 2, bottom row plots). Activity profiles confirmed the completely eliminated activity in the left V1 after muscimol-injections (Fig. 2A, B). In the right V1, the profiles also clearly revealed the reduction of ipsilateral eye evoked activities: the peak activity was reduced by 24% from 1.69±0.18 to 1.28±0.20 after muscimol-injection. A more pronounced reduction of ipsilateral evoked activity at lateral V1-regions, i.e. at the V1/V2 border, and as described for rat V1 [8] was not observed.

## Discussion

Here we demonstrated that inactivating one hemisphere in adult mouse V1 significantly reduced ipsilateral eye evoked activity in the entire binocular part of V1 of the opposite hemisphere. These data emphasize that also in adult mouse V1, cortico-cortical interactions play a major role for determining ocular dominance, and thus significantly extend previous knowledge about callosal influences on binocularity in juvenile rats [8], [9], [10]. Our data are also in line with recent extracellular single unit recordings in PD90 mice showing that the ipsilateral eye's response was reduced by removing the callosal input [10]. Here we demonstrate that the enhancing effect of cortico-cortical interactions on visually driven ipsilateral eye activity can also be observed in the population response, i.e. is a rather general influence, and also extends over the entire binocular part of V1.

We acutely silenced one hemisphere with intracortical microinjections of muscimol [8], [9], [16] and recorded visually evoked activity simultaneously in both V1 using intrinsic signal optical imaging. Optical imaging is ideally suited to verify the total block of activity in the entire V1 of the injected hemisphere and at the same time to visualize activity changes in the opposite, non-injected hemisphere across the entire activated V1. Recent studies in rats during the critical period for OD plasticity have revealed an important contribution of cortico-cortical communication to ocular dominance and its plasticity using extracellular action potential and visual evoked potential (VEP) recordings at the vertical midline representation in V1 [8], [9]. Injections of TTX into the ipsilateral LGN strongly reduced the contralateral eye evoked VEP-amplitude (about 80% reduction), whereas the ipsilateral eye evoked VEP was less reduced (30%: [9]. The authors concluded that the ipsilateral eye input is partially routed via the contralateral LGN and callosum. In monocularly deprived young rats, the callosum contributed to the decrease of the deprived, contralateral eye afferents in V1 [8]. Consistent with data from juvenile rat and adult mice [9], [10], here, acute blockade of activity in one hemisphere resulted in a decrease of ipsilateral eye evoked activity in V1 of the opposite hemisphere. Thus, we observed a global and excitatory net effect of the contralateral cortex on the ipsilateral eye evoked activity in the right V1 while contralateral eye evoked activity was not influenced.

Even though cortico-cortical interactions contribute a considerable part of the ipsilateral eye input to V1, still most ipsilateral input arrives through the direct thalamocortical pathway [8], [17], raising the interesting question about the function of this dual ipsilateral eye input. Our findings in adult mice and previously published single unit data [10] corroborate observations in young rats showing that silencing the ipsilateral LGN with TTX reduced the ipsilateral eye evoked VEP-amplitude in V1 and prolonged its latency thus uncovering an ipsilateral eye input via the callosum [9]. However, the results from juvenile rats are difficult to reconcile with other findings in young mice demonstrating a complete block of afferent field EPSPs in V1 after inactivation of the ipsilateral LGN with muscimol, and thus ruling out a contribution of callosally mediated input from the ipsilateral eye [18]. It would be interesting to investigate whether a later maturation of callosal ipsilateral eye input in mice could explain the different findings.

While both the previous data in juvenile rat and our present observation in adult mice report a strong influence of cortico-cortical projections on ocular dominance [8], [9], the spatial distribution of the observed effect seems to differ in both datasets. It was hypothesized that the denser callosal innervation near the midline [17], [19] results in a stronger effect of callosal inputs in lateral V1 representing the midline [8]. In our experiments, we therefore expected a medial shift of V1-activity after muscimol-inactivation of the opposite hemisphere. However, our imaging experiments did not provide evidence for this. In fact, callosal input in mice and rats extends across the whole V1 including the monocular region. While tracer and degeneration studies in mice and rats showed a band with high density of callosal cells in layers 2, 3 and 5 and callosal afferents in all layers at the area 17/18a border, a smaller number of cells and callosal afferents are also present throughout area 17 in layers 5 and 6 [19], [20], [21], [22], [23], [24]. Therefore, in rats and mice, callosal connections are not restricted to the midline and also connect sites representing locations at opposite directions in visual space [25]. These connections representing the visual periphery might serve completely different functions than connections at the midline and within the binocular part of V1 (reviewed in [26]. In addition, the effect of callosal inputs depends on the specific stimulus used (reviewed in [27].

Despite the attractiveness of the callosal pathway as the most straightforward explanation of the observed effect of muscimol-inactivation on ipsilateral eye input to V1, other sources must also be considered given the existence of massive descending and feedback projections connecting the cortex and thalamus. Traditionally, pathways connecting the cortex and the thalamus have been considered as unilateral reciprocal loops, although bilateral projections were described in e. g. the motor cortex [28], [29], [30], [31], [32], [33]. For the cat visual cortex, descending bilateral projections to the thalamus have been shown with large tracer injections [34]. Therefore contralateral cortico-thalamo-cortical pathways may mediate interhemispheric communication.

Other pathways which could mediate interhemispheric influences are bilateral connections of the thalamic reticular nuclei and thalamic nuclei [35], [36], [37]. Reversibly silencing the somatosensory barrel field with muscimol resulted in instantaneous, bilateral changes of sensory responses in the thalamus [38]. A role of these interhemispheric subcortical pathways has been discussed in connection with activity changes after stroke, which is a rather localized manipulation compared to total blockage of one hemisphere. Acute stroke damage of the somatosensory forelimb cortex in mice resulted in immediate changes of somatosensory responses in the unaffected, contra-stroke hemisphere and this was also observed in acallosal mice [39].

Cortical projections to primary sensory thalamic nuclei arise mainly from layer 6 neurons, which also project to more superficial layers of the cortex (reviewed in [40], [41], [42], [43]). Therefore layer 6 neurons can modulate sensory cortical responses directly via intracortical projections and indirectly via a cortico-thalamo-cortical loop. Optogenetic manipulation of the discharges of layer 6 neurons while simultaneously recording extracellulary in dLGN and visual cortex *in vivo* [44] showed that the influence of layer 6 neurons on the visual cortex was largely explainable with intracortical circuits, despite a minor influence on the dLGN. How contralateral cortico-thalamo-cortical circuits might modulate cortical responses and how they might interact with direct cortico-cortical influences via the callosum originating from layer 2,3 and 5 [23] remains to be determined. Furthermore, cortico-thalamo-cortical pathways or bilateral thalamic or reticulothalamic connections must be eye-specific in order to play a role in mediating the decreased V1-responses of the ipsilateral eye after silencing the contralateral cortex described here.
